# Interleukin-1-Interleukin-17 Signaling Axis Induces Cartilage Destruction and Promotes Experimental Osteoarthritis

**DOI:** 10.3389/fimmu.2020.00730

**Published:** 2020-05-05

**Authors:** Hyun Sik Na, Jin-Sil Park, Keun-Hyung Cho, Ji Ye Kwon, JeongWon Choi, Jooyeon Jhun, Seok Jung Kim, Sung-Hwan Park, Mi-La Cho

**Affiliations:** ^1^The Rheumatism Research Center, Catholic Research Institute of Medical Science, College of Medicine, The Catholic University of Korea, Seoul, South Korea; ^2^Department of Orthopedic Surgery, Uijeongbu St. Mary’s Hospital, College of Medicine, The Catholic University of Korea, Seoul, South Korea; ^3^Division of Rheumatology, Department of Internal Medicine, Seoul St. Mary’s Hospital, College of Medicine, The Catholic University of Korea, Seoul, South Korea; ^4^Department of Medical Lifescience, College of Medicine, The Catholic University of Korea, Seoul, South Korea; ^5^Department of Biomedicine and Health Sciences, College of Medicine, The Catholic University of Korea, Seoul, South Korea

**Keywords:** osteoarthritis, inflammation, interleukin-17, IL-1 receptor antagonist knockout, intestinal homeostasis

## Abstract

Osteoarthritis (OA), which is the most common degenerative joint disorder, has been considered a non-inflammatory disease with abnormal mechanics. Interleukin (IL)-17 is a pleiotropic cytokine involved in inflammatory diseases and their production is driven by the cytokine including IL-1 and IL-23. However, little is known about the mechanism of IL-17 in the development of OA. Here, we investigated the role of IL-17 in the pathogenesis of OA using monosodium iodoacetate (MIA)-injected IL-17 and IL-1 receptor antagonist (IL-1Ra) double-deficient mice. In MIA-injected IL-1Ra KO mice, nociceptive properties, degree of cartilage damage, and the level of inflammatory factors in articular cartilage were increased compared to MIA-injected wild-type mice. Interestingly, the intestinal architecture was impaired in IL-1Ra KO mice compared to wild-type mice and the damage was further exacerbated by MIA injection. Deficiency of IL-17 reduced nociceptive properties and cartilage destruction, as well as inflammation-related factors in MIA-injected IL-1Ra KO mice compared to MIA-injected wild-type mice. Furthermore, IL-17-treated chondrocytes from OA patients showed enhanced expression of catabolic factors that are involved in the destruction of cartilage in OA. IL-17 accelerates the destruction of cartilage and small intestine via regulation of several inflammatory mediators in an OA murine model. These results suggest that IL-17 plays a critical role in the development of OA.

## Introduction

Osteoarthritis (OA) is an age-related degenerative joint disorder characterized by progressive cartilage destruction, osteophyte formation, subchondral bone remodeling, and substantial functional disability and pain in the aged population (1). Among these manifestations, cartilage homeostasis disorder is a hallmark of OA caused by degradation of extracellular matrix such as proteoglycan and type II collagen through the actions of matrix-degrading enzymes including matrix metalloproteinase 3 (MMP3), MMP13, and ADAMTS5 (2–4). Although previous studies have identified factors or signaling pathways responsible for the progress of OA, there are no available disease-modifying OA drugs (DMOAD) and current medication is very limited to reduce pain. Therefore, there is an urgent unmet medical need for novel therapeutic approaches that can be used to improve the symptoms of OA.

Osteoarthritis has been considered a non-inflammatory disease focused on abnormal mechanics. However, recent findings suggest that inflammation plays a crucial role and is actively involved in the generation of joint symptoms and the progression of disease (5–7). Among various inflammatory mediators, proinflammatory cytokines play important roles in the development of OA (8). The level of tumor necrosis factor (TNF)-α and interleukin (IL)-6 increases in cartilage or synovial fluid (9, 10). Furthermore, IL-1 is positively correlated with osteoarthritic changes and directly contributes to cartilage destruction by affecting the balance of catabolic and anabolic factors for cartilage (8, 11).

IL-17 is a pleiotropic cytokine, which is mainly secreted by IL-17-producing T helper (Th17) cells and mast cells, and is implicated in autoimmune diseases including rheumatoid arthritis, inflammatory bowel disease, and multiple sclerosis (12, 13). IL-17 has a protective role in intestinal homeostasis, which regulates intestinal barrier integrity by controlling cellular localization of the tight junction protein occluding (14). However, IL-17, which is released from the intestine when it is damaged, plays a pathogenic role. The production of IL-17 is driven by the cytokine including IL-6, IL-23, and IL-1 (15, 16). It has been reported that deficiency of IL-1 receptor antagonist (IL-1Ra), endogenous inhibitor of IL-1, promotes the marked induction of IL-17 (17). Serum levels of IL-17A are statistically higher in OA patients than in healthy controls (18) and synovial fluid IL-17 concentration increases with Kellgren and Lawrence grade and positively correlates with Lequesne index in OA patients (19, 20). IL-17 induces the production of IL-8, growth-related oncogene alpha (GRO-α), and monocyte chemoattractant protein 1 (MCP-1) in chondrocytes (21). However, little is known about the role of IL-17 in the development of OA.

In this study, we investigated the role of IL-17 in the development of OA using IL-17-overexpressed IL-1Ra knockout (KO) mice and IL-17-deficient IL-1Ra KO mice (IL-1Ra and IL-17 double-deficient mice). In MIA-injected IL-1Ra KO mice, nociceptive properties and the degree of cartilage damage were increased compared to MIA-injected wild-type mice, whereas deficiency in IL-17 reduced these, as well as the expression of inflammation-related factors. Furthermore, IL-17-treated chondrocytes from OA patients showed enhanced expression of catabolic factors involved in the destruction of cartilage in OA.

## Materials and Methods

### Animals

Male BALB/c mice (8 weeks old) weighing 18–22 g at the start of the experiment were purchased from Orient Bio (Seongnam, South Korea). IL-1Ra KO and IL-17 KO mice were obtained from Prof. Yoichiro Iwakura (University of Tokyo, Japan). IL-1Ra KO mice were backcrossed with IL-17 KO mice over 10 generations, and IL-1Ra and IL-17 double-deficient mice were selected for use in polymerase chain reaction (PCR) analyses. The animals were maximally housed at five per cage in a room with controlled temperature (20–26°C) and lighting (12 h light–dark cycle) with access to gamma ray sterilized diet (TD 2018S, Harlan Laboratories, Inct/America) and autoclaved R/O water. All animal research procedures were provided in accordance with the Laboratory Animals Welfare Act, the Guide for the Care and Use of Laboratory Animals, and the Guidelines and Policies for Rodent experiments provided by the Department of Laboratory Animals, Institutional Animal Care and Use Committee (IACUC), the Catholic University of Korea (CUMC-2017-0193-04), conforming to all National Institutes of Health (United States) guidelines. IACUC and the Department of Laboratory Animal (DOLA) at Catholic University of Korea, Songeui Campus, accredited the Korea Excellence Animal laboratory Facility from Korea Food and Drug Administration in 2017 and acquired AAALAC International full accreditation in 2018.

### Monosodium Iodoacetate Model of OA

**FIGURE 1:**
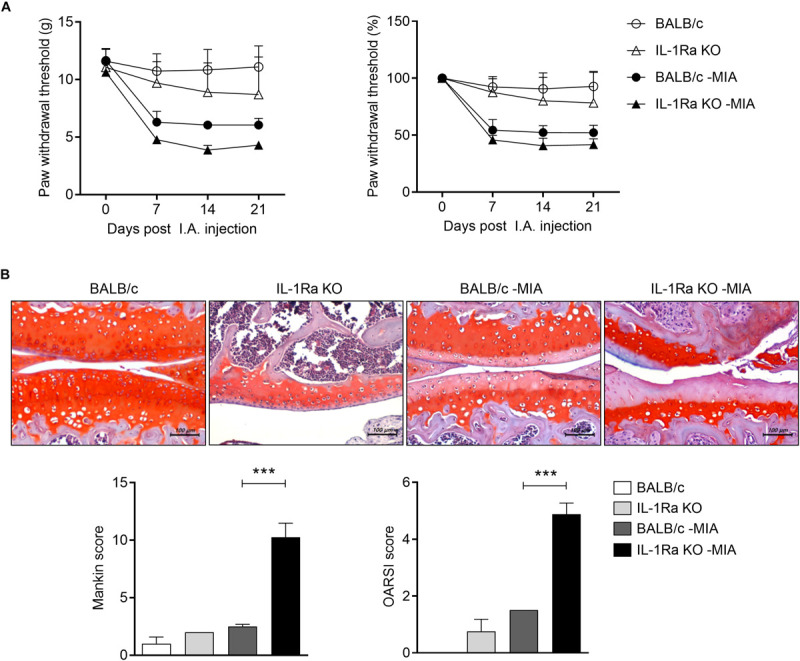
IL-1Ra deficient mice injected with MIA are more sensitive to pain and the treatment promotes articular cartilage damage. **(A)** BALB/c and IL-1Ra KO mice were injected intra-articularly with 0.6 mg MIA in the right knee. Behavioral tests of secondary tactile allodynia in MIA-injected BALB/c and IL-1Ra KO mice and untreated BALB/c and IL-1Ra KO mice were evaluated using a dynamic plantar esthesiometer (BALB/c *n* = 5, IL-1Ra KO *n* = 4, BALB/c MIA *n* = 4, IL-1Ra KO MIA *n* = 3). **(B)** At 3 weeks after the MIA injection, sections of articular tissue from mice were stained with Safranin O and then we evaluated the severity of Mankin and OARSI scores. Representative histological features are shown (original magnification 200×). Three independent experiments were performed. Data are shown as means ± SDs. ***P < 0.001 vs. MIA-injected BALB/c group (One-way ANOVA followed by Bonferroni *post hoc* test).

**FIGURE 2:**
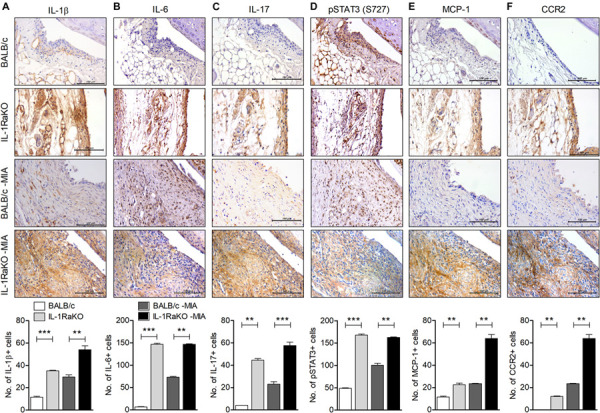
IL-1Ra deficiency upregulates the level of inflammatory factors in joints of an MIA-induced murine model. BALB/c and IL-1Ra KO mice were injected intra-articularly with 0.6 mg MIA in the right knee. At 3 weeks after the MIA injection, sections of articular tissue from MIA-injected mice and untreated BALB/c and IL-1Ra KO mice were immunostained for IL-1β **(A)**, IL-6 **(B)**, IL-17 **(C)**, phosphorylated STAT3 (S727) **(D)**, MCP-1 **(E)**, and CCR2 **(F)**. Representative histological features are shown (original magnification 400×). The results are quantified as the means ± SDs for number of antibody-positive cells in three animals per group. **P < 0.01, ***P < 0.001. BALB/c vs. IL-1Ra KO. MIA-injected BALB/c group vs. MIA-injected IL-1Ra KO group (One-way ANOVA followed by Bonferroni *post hoc* test).

**FIGURE 3:**
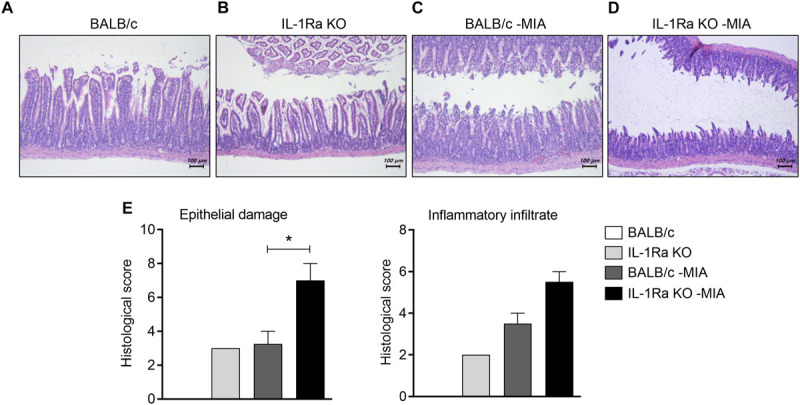
IL-1Ra deficiency aggravates the histological structure of the small intestine in an MIA-injected OA model. **(A–E)** BALB/c and IL-1Ra KO mice were injected intra-articularly with 0.6 mg MIA in the right knee. At 3 weeks after the MIA injection, sections of small intestine from MIA-injected mice and untreated BALB/c and IL-1Ra KO mice were stained with hematoxylin and eosin. Representative histological features are shown (original magnification 100×). **(E)** The graph shows the histological and inflammatory infiltrates score quantified. Data are means ± SDs. *P < 0.05. MIA-injected BALB/c group vs. MIA-injected IL-1Ra KO mice group (2-tailed *t*-test).

**FIGURE 4:**
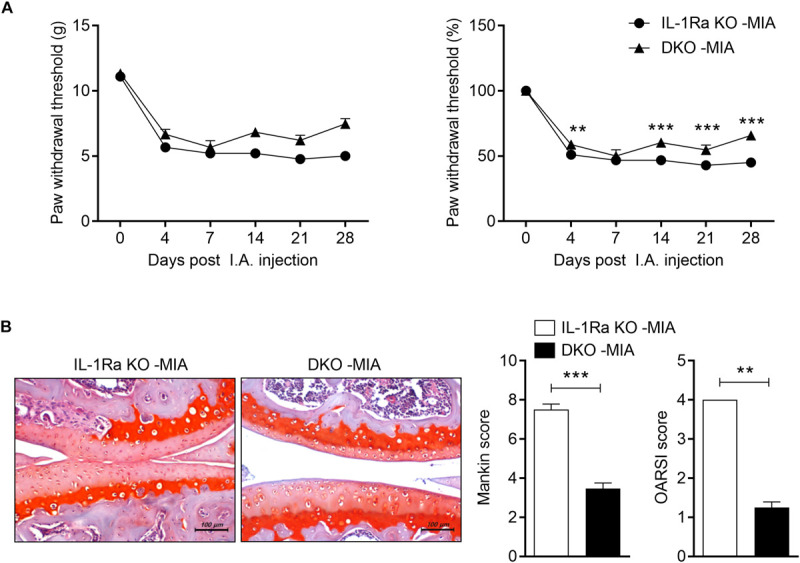
IL-17 deficiency ameliorates pain and cartilage damage. IL-1Ra KO mice and IL-17-deficient IL-1Ra double-deficient mice (DKO) were injected with 0.6 mg MIA in the right knee. **(A)** Behavioral tests of secondary tactile allodynia in MIA-injected IL-1Ra KO mice and IL-1Ra and IL-17 double-deficient mice were evaluated using a dynamic plantar esthesiometer (IL-1Ra KO MIA *n* = 3, DKO MIA *n* = 4). Experimental mechanical pain was analyzed using the PWT. **(B)** At 4 weeks after the MIA injection, sections of articular tissue from mice were stained with Safranin O and we evaluated the severity of Mankin and OARSI scores. Representative histological features are shown (original magnification 200×). Three independent experiments were performed. Data are means ± SDs. **P < 0.001, ***P < 0.001 MIA-injected IL-1Ra KO mice group vs. MIA-injected DKO [2-tailed *t*-test **(B)**].

**FIGURE 5:**
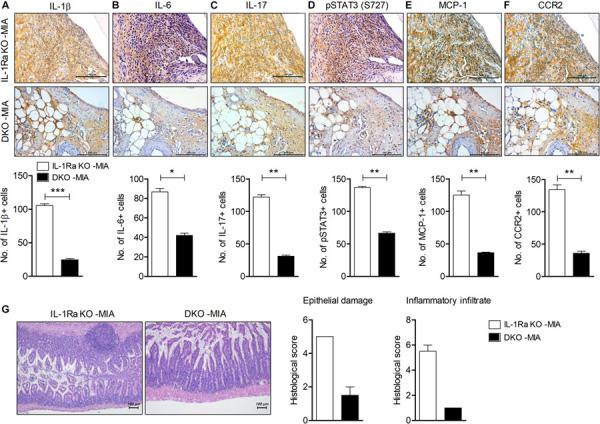
IL-17 deficiency downregulates inflammation in cartilage and intestine in MIA-injected IL-1Ra KO mice. IL-1Ra KO mice and IL-17-deficient IL-1Ra KO mice (DKO) were injected with 0.6 mg MIA in the right knee. At 4 weeks after the MIA injection, articular tissues and small intestine from each group of mice were isolated. **(A–F)** Sections of articular tissue from mice were immunostained for IL-1β **(A)**, IL-6 **(B)**, IL-17 **(C)**, phosphorylated STAT3 (S727) **(D)**, MCP-1, **(E),** and CCR2 **(F)**. Representative histological features are shown (original magnification 400×). The results are quantified as means ± SDs for number of antibody-positive cells in three animals per group. **(G)** Sections of small intestine from mice were stained with hematoxylin and eosin. Representative histological features are shown (original magnification 100×). The graph shows the histological and inflammatory infiltrates score quantified. Data are means ± SDs. *P < 0.05, **P < 0.01, ***P < 0.001 vs. MIA-injected IL-1Ra KO mice group (2-tailed *t*-test).

Osteoarthritis was induced through a single intra-articular injection of MIA. After anesthesia with 2% isoflurane, 8-week-old male BALB/c (*n* = 4) and IL-1Ra KO (*n* = 3) mice in Figures 1–3 and IL-1Ra KO (*n* = 3) and IL-17 and IL-1Ra double-deficient (*n* = 4) mice in Figures 4, 5 were injected intra-articularly with 0.6 mg monosodium iodoacetate (MIA) (Sigma, United States) in a 20 μL into the right knee via a Hamilton syringe (22); control mice were injected with an equivalent volume of saline. Mice underwent testing to measure nociceptive threshold on days 0, 7, 14, 21, or 28 after injection of MIA or saline. Animals were sacrificed on day 21 or 28 after MIA injection. Three independent experiments were performed.

### Assessment of Pain

Joint nociception was evaluated using a von Frey anesthesiometer (IITC, United States), which is essentially an electronic version of the von Frey assessment procedure used to assess mechanical sensitivity. Mice were placed on a metal mesh surface in an acrylic chamber in a temperature-controlled room (20–26°C) and rested for 10 min before testing. The plantar surface of mice was vertically stimulated with anesthesiometer. The force required to elicit a paw-withdrawal reflex was recorded automatically and measured in grams. A maximum force of 90 g was applied. All procedures and testing were performed in a blinded manner.

### Histopathological Analyses

Joints and small intestines were collected from each group at 3–4 weeks post-MIA. Tissues were fixed in 10% formalin solution and decalcified using Calci-Clear solution (National Diagnostics, United States). Tissues were embedded in paraffin and sectioned at 4–5 μm thickness, dewaxed using xylene, dehydrated through an alcohol gradient, and stained with Safranin O and hematoxylin and eosin (H&E), respectively. Cartilage damage was scored as described previously (23, 24). Loss of epithelium, crypt damage, depletion of goblet cells, and infiltration of inflammatory cells were assessed in these sections as described previously (25).

### Immunohistochemistry

Paraffin samples were incubated at 4°C with the primary monoclonal antibodies (Abs) IL-1β, IL-17, MCP-1, phosphorylated signal transducer and activator of transcription 3 (STAT3) (S727) (Abcam, United Kingdom), IL-6, and C-C chemokine receptor type 2 (CCR2) (Novus Biologicals, United States). Then the samples were incubated with the respective secondary antibodies. The primary antibodies were detected with a biotinylated secondary linking Ab, followed by incubation with streptavidin–peroxidase complex for 30 min. The product was developed using 3, 3-diaminobenzidine chromogen (Dako, United States). The positive cells were enumerated visually by four individuals and the mean values were calculated.

### Isolation of Human Chondrocytes and Stimulation

Articular cartilage for human was acquired from patients to replacement arthroplasty or joint replacement surgery (*n* = 2) (UC14CNSI0150). One patient has a KL equal to 3 and the other one to 4, as determined by scoring of the patient’s x-ray radiographs by an orthopedic surgeon prior to surgery. Cartilage obtained from the patient was digested and reacted with 0.5 mg/mL hyaluronidase, 5 mg/mL protease type XIV, and 2 mg/mL collagenase type V. The isolated chondrocytes were seeded in 24-well plates at 2 × 10^4^ cells/well and treated with IL-17 (10 and 50 ng/mL) for 24 or 48 h.

### Real-Time Polymerase Chain Reaction (PCR)

Messenger RNA (mRNA) was isolated from human chondrocytes using the TRI reagent (Molecular Research Center, United States) and complementary DNA was synthesized from the RNA. A LightCycler 2.0 instrument (Roche Diagnostics, software version 4.0) was used for PCR amplifications. Relative expression of specific mRNA was quantified by real-time PCR using SensilFAST SYBR (Bioline, United States). The following sense and antisense primers were used: for MMP1, 5′-CTG AAG GTG ATG AAG CAG CC-3′ (sense) and 5′-AGT CCA AGA GAA TGG CCG AG-3′ (anti-sense); for MMP3, 5′-CTC ACA GAC CTG ACT CGG TT-3′ (sense) and 5′-CAC GCC TGA AGG AAG AGA TG-3′ (anti-sense); for MMP13, 5′-CTA TGG TCC AGG AGA TGA AG-3′ (sense) and 5′-AGA GTC TTG CCT GTA TCC TC-3′ (anti-sense); for MCP-1, 5′-CAG CCA GAT GCA ATC AAT GC-3′ (sense) and 5′-GTG GTC CAT GGA ATC CTG AA-3′ (anti-sense); for tissue inhibitor of metalloproteinase-3 (TIMP3), 5′-CTG ACA GGT CGC GTC TAT GA-3′ (sense) and 5′-GGC GTA GTG TTT GGA CTG GT-3′ (anti-sense); for collagen type II alpha 1 chain (COL2A1), 5′-TCT ACC CCA ATC CAG CAA AC-3′ (sense) and 5′-GTT GGG AGC CAG ATT GTC AT-3′ (anti-sense); for SOX9, 5′-ACT TGC ACA ACG CCG AG-3′ (sense) and 5′-CTG GTA CTT GTA ATC CGG GTG-3′ (anti-sense). The mRNA levels were normalized to that of β-actin mRNA.

### Statistical Analyses

Statistical analyses were performed using GraphPad Prism software (version 5 for Windows). Experimental values are presented as means ± standard deviations. *P*-values were calculated with two-tailed *t*-tests and two-way ANOVA analyses of variance (grouped). *P*-values < 0.05 (two-tailed) were considered to indicate statistical significance.

## Results

### IL-1Ra Deficiency Accelerates Pain and Cartilage Destruction in the MIA-Injected Murine Model

The pathogenic role of IL-17 has been investigated on a model expressing abnormal level of IL-1 and IL-17 caused by IL-1Ra deficiency. IL-1Ra KO mice were injected with MIA IA and secondary tactile allodynia was assessed using the automated von Frey hair assessment system. The paw withdrawal threshold (PWT) was decreased in MIA-injected IL-1Ra KO mice compared to MIA-injected BALB/c mice (Figure 1A). Interestingly, the nociceptive response in IL-1Ra KO was similar to that of MIA-injected BALB/c mice. To determine the degree of cartilage degradation of OA joints, knees were isolated and the cartilage was stained with Safranin O (Figure 1B). Joint cartilage morphology was quantitatively evaluated using the modified Mankin’s scoring system, which assesses parameters such as structural changes, cellular abnormalities, and matrix staining, and the OARSI scoring system, which consists of a grading and staging component on structural integrity of the articular cartilages. Mankin and OARSI scores revealed significant cartilage thickness and depletion of proteoglycan in IL-1Ra KO mice injected with MIA compared to MIA-injected BALB/c mice (*P* < 0.001, One-way ANOVA followed by Bonferroni *post hoc* test).

### IL-1Ra Deficiency Increases the Level of Inflammatory Factors in the Cartilage of the MIA-Injected Murine Model

To determine the degree of inflammatory mediators in IL-1Ra-deficient environments, the levels of IL-1β, IL-6, and IL-17 in cartilage from BALB/c and IL-1Ra KO mice and the MIA-injected murine model was measured via immunohistochemistry. Levels of IL-1β, IL-6, and IL-17 were highly expressed in cartilage of IL-1Ra KO mice compared to BALB/c mice (*P* < 0.001, *P* < 0.001, *P* < 0.01, respectively; One-way ANOVA followed by Bonferroni *post hoc* test) (Figures 2A–C). To explore the effects of these increases in inflammatory cytokines on STAT3 phosphorylation, phosphorylated STAT3 (S727) was immunostained in the cartilage of each group (Figure 2D). As expected, the level of phosphorylated STAT3 (S727) was already high in IL-1Ra KO mice (*P* < 0.001, One-way ANOVA followed by Bonferroni *post hoc* test). MCP-1, a chemoattractant molecule, is expressed in the synovial sublining cells of the OA joint and binds to multiple receptors, but most strongly binds to the cell surface leucocyte receptor CCR2 (26, 27). Recently, the MCP-1 and CCR2 signaling axis was found to regulate neuropathic pain and neuroinflammation and play a role in pain-related behaviors in post-traumatic murine OA (28, 29). In our study, the expressions of MCP-1 and CCR2 were significantly higher in the cartilage tissues of MIA-injected IL-1Ra KO mice than in BALB/c injected with MIA (*P* < 0.001, One-way ANOVA followed by Bonferroni *post hoc* test) (Figures 2E,F).

### Progression of OA Exacerbates Intestinal Inflammation in MIA-Injected IL-1Ra KO Mice

To explore the effects of IL-1Ra deficiency on intestinal inflammation, small intestine tissues from BALB/c and IL-1Ra KO mice were stained with H&E (Figure 3). Compared to BALB/c mice (Figure 3A), the intestinal architecture was severely impaired and inflammatory infiltrates increased in the intestines of IL-1Ra deficient mice (Figure 3B). MIA-injected BALB/c mice (Figure 3C) showed more damage to the mucosal architecture than BALB/c mice (Figure 3D). Particularly, the number of infiltrated cells and histological scores (*P* < 0.05, 2-tailed *t*-test) were higher in MIA-injected IL-1Ra KO (Figure 3E).

### IL-17 Exacerbates the Nociceptive Response and Cartilage Damage in the MIA-Injected IL-1Ra KO Model

To explore whether increased levels of IL-17 promote OA development in MIA-injected IL-1Ra KO mice, we constructed IL-1Ra and IL-17 double-deficient mice. In the von Frey hair assessment test, PWT results were increased in MIA-injected IL-1Ra and IL-17 double-deficient mice compared to MIA-injected IL-1Ra KO mice (Figure 4A). Furthermore, IL-17-deficient IL-1Ra KO mice injected with MIA had significantly lower Mankin and OARSI scores than the IL-1Ra KO mice injected with MIA (*P* < 0.001, *P* < 0.01, respectively; 2-tailed *t*-test) (Figure 4B). IL-17 deficiency prevented cartilage damage and cellular abnormalities. These results indicate that IL-17 plays an important role in the progression of OA.

### IL-17 Augments Inflammation in the Cartilage and Intestine of MIA-Injected IL-1Ra KO Mice

To determine the effects of IL-17 deficiency on joint inflammation in an OA model injected with MIA into IL-1Ra KO mice, we performed immunohistochemical staining for inflammation and pain-related mediators in the cartilage (Figure 5). IL-17 expression was significantly lower and the number of cells expressing IL-1β, IL-6, and phosphorylated STAT3 (S727) was significantly reduced in MIA-injected IL-1Ra and IL-17 double-deficient mice compared to IL-1Ra KO mice injected with MIA (*P* < 0.01, *P* < 0.001, *P* < 0.05, *P* < 0.01, respectively; 2-tailed *t*-test) (Figures 5A–D). Furthermore, the expressions of MCP-1 and CCR2 were also markedly reduced in the cartilage of MIA-injected IL-1Ra and IL-17 double-deficient mice compared to MIA-injected IL-1Ra KO mice (*P* < 0.01, 2-tailed *t*-test) (Figures 5E,F); their degree of intestinal inflammation was also reduced (Figure 5G). These results suggest that IL-17 plays a key role in the development of OA by increasing pathogenic mediators.

### IL-17 Regulates the mRNA Expression of Catabolic and Anabolic Factors

**FIGURE 6:**
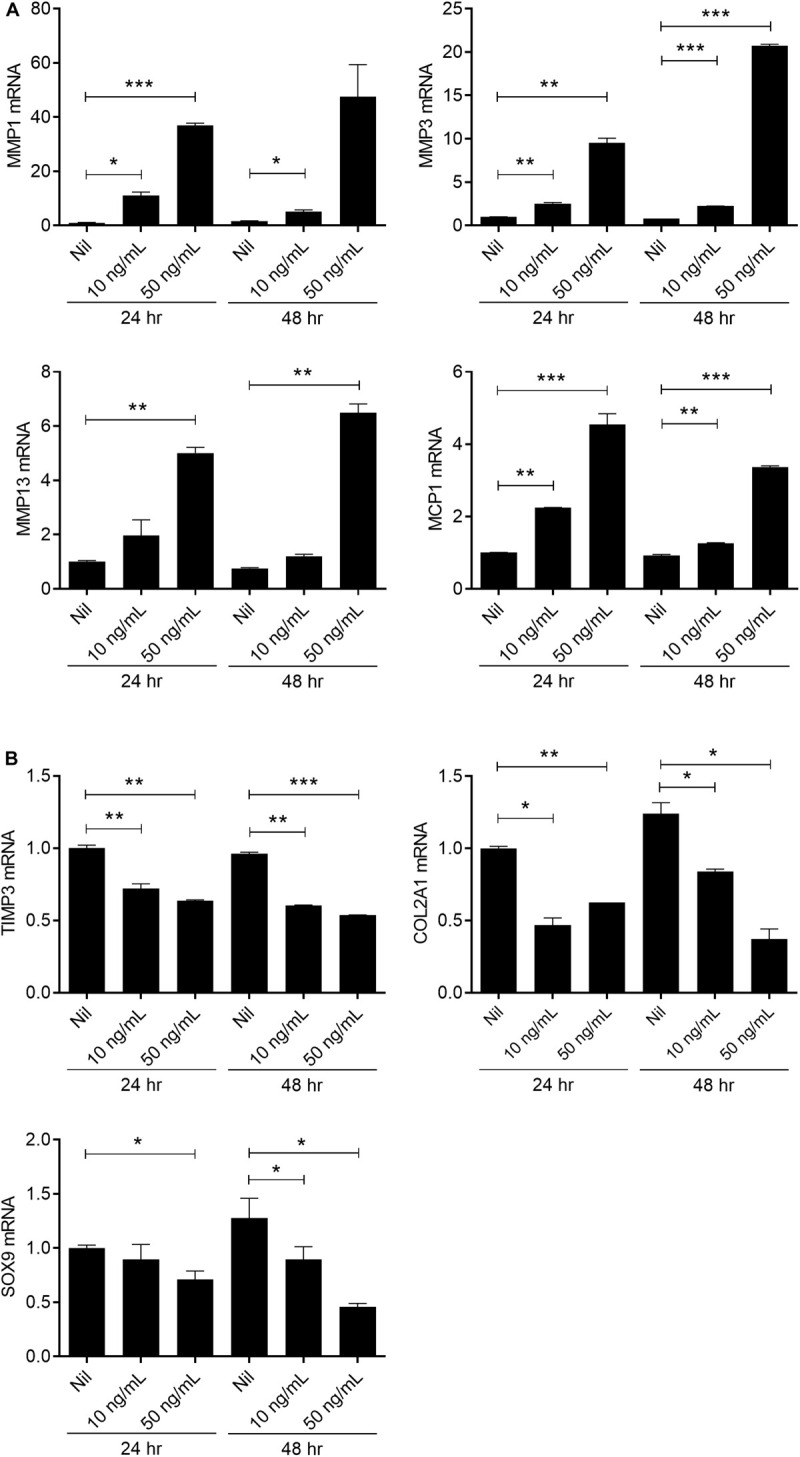
IL-17 promotes catabolic activity in chondrocytes from OA patients. Human articular chondrocytes from OA patients (*n* = 2) were stimulated with IL-17 (10, 50 ng/mL) for 24 h or 48 h. Expression levels of catabolic markers (MMP 1, 3, 13) **(A)**, chemokine MCP-1, anabolic marker (TIMP3), and chondrogenesis markers (COL2A1 and SOX9) **(B)** were analyzed by real-time PCR. Data are representative of three independent experiments with similar results. Data are shown as the means ± SDs. *P < 0.05, **P < 0.01, ***P < 0.001 vs. Nil (2-tailed *t*-test).

The balance of catabolic and anabolic activity is important in maintaining the integrity of cartilage tissue. Increases in the amount of matrix-degradative enzymes, such as MMPs, cause an imbalance between the synthesis and degradation of matrix elements, resulting in excessive tissue destruction (30, 31). To explore the effects of IL-17 on chondrogenesis, human chondrocytes were stimulated with IL-17 and then we evaluated the expression levels of catabolic, anabolic, and chondrogenesis factors. The expressions of MMP1, 3, 13, and MCP-1 were significantly increased (2-tailed *t*-test), whereas the expressions of TIMP3 and COL2A1 were significantly decreased by IL-17 dose-dependently (2-tailed *t*-test). In addition, the expression of SOX9, a master anabolic chondrogenic transcription factor (32), was decreased in an IL-17-dependent manner (2-tailed *t*-test) (Figure 6). These results suggest that IL-17 is a key promoter of cartilage destruction.

## Discussion

We focused on the pathogenic roles of IL-17 in the progression of OA using a murine OA model. IL-1Ra KO mice showed higher levels of nociceptive properties and degrees of cartilage damage than WT, and IL-1Ra KO mice were more susceptible to the development of disease even when OA disease was induced by MIA injection. In addition, compared to WT, IL-1Ra KO mice showed severe destruction of the intestinal structure and increased inflammation. However, IL-17-deficient IL-1Ra KO mice injected with MIA showed decreased pain and cartilage damage as well as inflammation in the intestines compared to IL-1Ra KO mice injected with MIA. Furthermore, IL-17-treated chondrocytes from OA patients showed enhanced expression of catabolic factors that are involved in the destruction of cartilage in OA.

Osteoarthritis has been considered a non-inflammatory and degenerative disease, but in recent studies, the role of inflammation in OA initiation and progression has been supported and inflammation is considered important in disease progression (5–7). IL-17, which is mainly produced in Th17 cells, promotes cartilage degradation by promoting MMPs, disintegrin, and metalloproteinase, with thrombospondin motifs and inducible nitric oxide synthase, similar to key inflammatory cytokines such as IL-6, TNF-α, and IL-1β (8). IL-17 levels are elevated in the serum and the synovial fluid of patients with OA and are positively correlated with OA severity. In addition, IL-17 induces the production of IL-8, GRO-α, and MCP-1 in chondrocytes (19, 21) and stimulates fibroblasts and chondrocytes in OA to promote the secretion of cytokines such as TNF-α, IL-6, and IL-1β, and to further stimulate the production of cartilage-destroying factors and promote synovial infiltration (21, 33). On the other hand, it inhibits proteoglycan synthesis in murine and rat articular cartilage and inhibits extracellular matrix homeostasis (34, 35). Administration of DNA aptamers specific for IL-17 receptor inhibits IL-6 expression and synovial thickening in synovial tissues in a murine OA model (36).

There are numerous OA animal models to investigate the pathogenesis of OA and the therapeutic efficacy of new drugs. Surgically induced OA model is highly reproducible and progress rapidly and is suitable for study of the pathogenesis of post-traumatic osteoarthritis (37). Chemically induced OA model, cartilage is almost destructed in a short time, accompanied by inflammation. This chemical model represents a severe OA and joint replacement surgery patients (KL grade: 3∼4) (38). MIA, an inhibitor of glyceraldehyde-3-phosphate dehydrogenase activity, drives joint pathology mimicking that seen in human OA (KL grade 3∼4) (38). At the cellular level, MIA treatment induces apoptosis in chondrocytes via ROS production and mitochondria-mediated caspase-3 activation (39). Recently, several reports that study the anti-inflammatory effects of therapeutic agents in MIA-injected rats have been reported. SHINBARO, a refined herbal formulation, suppressed the induction of inflammation-mediated enzymes (iNOS and COX-2) and pro-inflammatory cytokines (TNF-α and IL-1β) via downregulation of NFkB in MIA-induced OA rat model (40). MIA injection in mice induced a transient increase in joint-related NFkB activity at early time and serum IL-6 levels were markedly increased on day 3 compared to control mice (41). These reports suggest that the MIA-injected rat model can be used to study OA pathogenesis. Further studies will be needed to confirm our hypothesis in other animal models suitable for OA pathogenesis study.

IL-1Ra is an endogenous inhibitor of IL-1 activity that competes with IL-1 (IL-1α and IL-1β) for binding to the IL-1 receptor. Iwakura and colleagues identified that IL-17 production is markedly induced in IL-1Ra KO mice and that the inflammatory arthritis in that animal model requires IL-17 and T cells (17). To investigate the role of IL-17 during the development of OA, we evaluated the severity of OA in MIA-injected IL-1Ra KO mice overexpressing IL-17 and IL-1Ra KO mice lacking IL-17 (IL-17 and IL-1Ra double-deficient mice). Our results showed that the nociceptive response and cartilage degradation worsened in IL-1Ra KO mice, whereas IL-17 deficient IL-1Ra KO showed decreased disease development. In addition, IL-17 stimulation increased the expression of catabolic factor and decreased the expression of anabolic factors. These results demonstrate that IL-17 plays a key role in OA development. However, there are some reports that IL-1 does not affect the pathological features of murine OA models. Deficiency of IL-1α or IL-1β is not involved in synovial inflammation, cartilage destruction, and the severity of OA in murine menisectomy model and collagen-induced OA model (42, 43). In our study, we examined the development of OA in the excess IL-1 and IL-17 circumstance caused by IL-1Ra deficiency; therefore, further studies are needed to elucidate the signaling network involved in IL-1-IL-17 axis.

There is growing evidence that perturbation of commensal intestinal microbiota is involved in the pathogenesis of various diseases including rheumatoid arthritis, obesity, and osteoporosis (44–46). Furthermore, changes in the gut microbiome and chronic systemic inflammation due to obesity can accelerate OA development (47, 48). Obesity-induced synovial inflammation, chondrocyte hypertrophy, and meniscal mineralization are mitigated through oligofructose, a non-digestible prebiotic fiber that facilitates the expansion of beneficial *Bifidobacteria* and improves insulin resistance (49). In addition, studies have investigated the therapeutic effects of OA by controlling gut microbiota through the administration of *Bifidobacterium longum* or prebiotic fiber supplementation (50, 51). Deletion of IL-1Ra changes the composition of intestinal microbial and decreases the diversity depending on toll-like receptor 4, and the production of IL-17 is significantly increased in the lamina propria lymphocytes of such mice; this indicates that IL-1Ra may regulate the diversity and composition of intestinal microbiota (52). In addition, in a previous study, SKG mice inoculated with a feces mixture obtained from RA patients showed a significant increase in arthritis development and response to autoantigen-reactive lymphocytes compared to SKG mice inoculated with a healthy feces mixture (53). Although these results have been demonstrated in autoimmune arthritis, dysbiosis can accelerate joint destruction by promoting an inflammatory response. In the present study, we showed that intestinal architecture and the degree of inflammation was deteriorated in IL-17-activated IL-1Ra KO mice. We found that intestinal architecture is severely impaired and inflammation and joint destruction are promoted in IL-17-overactivated IL-1Ra KO mice, while IL-17-deficient IL-1Ra KO mice lack these properties. These results suggest that increased nociceptive properties and increased severity of inflammation in IL-1Ra KO mice may be caused by an increase in IL-17 through an imbalance of the intestinal microbiome through IL-1Ra deficiency. Further studies are required to determine the effects of intestinal dysbiosis through IL-17 on joint destruction.

## Conclusion

In conclusion, our data are the first to demonstrate that IL-17 is a key factor in the development of OA using the MIA-injected IL-17-deficient IL-1Ra KO murine model. In IL-1Ra KO mice, administration of MIA accelerated the severity of disease compared to MIA-injected wild-type mice. However, IL-17 deficiency mitigated nociceptive properties and cartilage destruction as well as the expression of inflammation-related factors in MIA-injected IL-1Ra KO mice. These results suggest that IL-17 signaling axis has an indispensible role in the development of OA.

## Data Availability Statement

All datasets generated for this study are included in the article.

## Ethics Statement

The studies involving human participants were reviewed and approved by the Catholic University of Korea, Uijeongbu St. Mary’s Hospital (UC14CNSI0150). The patients/participants provided their written informed consent to participate in this study.

## Author Contributions

HN, J-SP, K-HC, SK, S-HP, and M-LC: conception and design of study. HN, K-HC, JK, and JC: acquisition of data. HN, J-SP, K-HC, JK, JC, and JJ: analysis and interpretation of data. J-SP, HN, and M-LC: drafting the article. J-SP and M-LC: revising the article critically. All authors have critically reviewed the manuscript and approved the final manuscript.

## Conflict of Interest

The authors declare that the research was conducted in the absence of any commercial or financial relationships that could be construed as a potential conflict of interest.
