# Corrigendum: Interleukin-1-Interleukin-17 Signaling Axis Induces Cartilage Destruction and Promotes Experimental Osteoarthritis

**DOI:** 10.3389/fimmu.2020.01862

**Published:** 2020-08-19

**Authors:** Hyun Sik Na, Jin-Sil Park, Keun-Hyung Cho, Ji Ye Kwon, JeongWon Choi, Jooyeon Jhun, Seok Jung Kim, Sung-Hwan Park, Mi-La Cho

**Affiliations:** ^1^The Rheumatism Research Center, Catholic Research Institute of Medical Science, College of Medicine, The Catholic University of Korea, Seoul, South Korea; ^2^Department of Orthopedic Surgery, Uijeongbu St. Mary's Hospital, College of Medicine, The Catholic University of Korea, Seoul, South Korea; ^3^Division of Rheumatology, Department of Internal Medicine, Seoul St. Mary's Hospital, College of Medicine, The Catholic University of Korea, Seoul, South Korea; ^4^Department of Medical Lifescience, College of Medicine, The Catholic University of Korea, Seoul, South Korea; ^5^Department of Biomedicine and Health Sciences, College of Medicine, The Catholic University of Korea, Seoul, South Korea

**Keywords:** osteoarthritis, inflammation, interleukin-17, IL-1 receptor antagonist knockout, intestinal homeostasis

In the original article, we neglected to include the funder “Ministry of Health & Welfare, Republic of Korea, HI15C1062” to author M-LC in the **Funding** statement.

The corrected statement is as follows:

“This research was supported by a grant of the Korea Health Technology R&D Project through the Korea Health Industry Development Institute (KHIDI), funded by the Ministry of Health & Welfare, Republic of Korea (HI15C1062), funded by the Ministry of Health & Welfare, Republic of Korea (HI15C3062), and Basic Science Research Program through the National Research Foundation of Korea (NRF) funded by the Ministry of Education (grant number NRF-2018R1D1A1B07048554).”

The authors apologize for this error and state that this does not change the scientific conclusions of the article in any way. The original article has been updated.

